# Supplementary material for: Developing a multi-institutional nomogram for assessing lung cancer risk in patients with 5–30 mm pulmonary nodules: a retrospective analysis
Source: PeerJ. 2023 Dec 13;11:e16539. doi: 10.7717/peerj.16539 (PMC10725170; doi:10.7717/peerj.16539)
Supplement: Supplemental Information 1 [file peerj-11-16539-s001.docx]

Status

0: Benign nodules; 1: Malignant nodules

Age

0: < 60 years old; 1: > = 60 years old

Gender

0: Man; 1: Woman

Annual smoking volume

0:﹤400 branch /year ;1: > = 400 branch /year

Density of the nodule

1= Pure ground-glass; 2= Mixed ground-glass; 3= Solid

Nodule diameter

0: < 10 mm; 1: 10-20 mm; 2: > 20 mm

Edge

0: Rough; 1: Smooth

Shape

0: Irregular; 1: Regular

Cavity

0: < 5 mm; 1: > = 5 mm

Other categorical data

0= Without corresponding element; 1= With corresponding element
