# Supplementary material for: Developing a multi-institutional nomogram for assessing lung cancer risk in patients with 5–30 mm pulmonary nodules: a retrospective analysis
Source: PeerJ. 2023 Dec 13;11:e16539. doi: 10.7717/peerj.16539 (PMC10725170; doi:10.7717/peerj.16539)
Supplement: Supplemental Information 4 [file peerj-11-16539-s004.docx]

data<-na.omit(data)

data$Age<-factor(data$Age,labels=c("<60",">=60"))

data$Gender<-factor(data$Gender,labels=c("Man","Woman"))

data$Smoking_history<-factor(data$Smoking_history,labels=c("NO","YES"))

data$Annual_smoking_volume<-factor(data$Annual_smoking_volume,labels=c("<400",">=400"))

data$Dust_exposure_history<-factor(data$Dust_exposure_history,labels=c("NO","YES"))

data$Family_history_of_malignancy<-factor(data$Family_history_of_malignancy,labels=c("NO","YES"))

data$Family_history_of_lung_cancer<-factor(data$Family_history_of_lung_cancer,labels=c("NO","YES"))

data$Density_of_the_nodule<-factor(data$Density_of_the_nodule,labels=c("Pure_ground_glass","Mixed_ground_glass","Solid"))

data$Nodule_diameter<-factor(data$Nodule_diameter,labels=c("<10","10-20",">20"))

data$Spiculation<-factor(data$Spiculation,labels=c("NO","YES"))

data$Edge<-factor(data$Edge,labels=c("Rough","Smooth"))

data$Lobulation<-factor(data$Lobulation,labels=c("NO","YES"))

data$Shape<-factor(data$Shape,labels=c("Irregular","Regular"))

data$Calcification<-factor(data$Calcification,labels=c("NO","YES"))

data$Cavity<-factor(data$Cavity,labels=c("<5",">=5"))

data$Vacuole<-factor(data$Vacuole,labels=c("NO","YES"))

data$Vascular_convergence<-factor(data$Vascular_convergence,labels=c("NO","YES"))

data$Bronchiole<-factor(data$Bronchiole,labels=c("NO","YES"))

data$Pleural_traction<-factor(data$Pleural_traction,labels=c("NO","YES"))
